# Depression in women recovered from COVID-19

**DOI:** 10.1192/j.eurpsy.2022.1245

**Published:** 2022-09-01

**Authors:** M. Lagha, G. Hamdi, N. Dhaouadi, S. Chebli, R. Ridha

**Affiliations:** 1 Razi Hospital, Forensic Psychiatry, La Manouba, Tunisia; 2 Abderrahmen Mami Hospital, Preventive Medicine, Ariana, Tunisia

**Keywords:** recovered, Depression, women, Covid-19

## Abstract

**Introduction:**

Studies assessing the psychological impact of COVID-19 have shown that patients with COVID-19 had significantly higher levels of depression, anxiety, and post-traumatic stress symptoms than healthy controls.

**Objectives:**

The objectives of our study were to assess depression in women recovered from COVID-19 and to compare it to healthy controls.

**Methods:**

It was a cross-sectional case-control study.

We randomly recruited women, from April 1st to 30th, 2021.The women in the case group have been infected with Sars-Cov 2, with a benign or pauci-symptomatic clinical form, and cured for one to two months at the time of the study without any post-COVID complications. Women included in the control group have not been infected with Sars-Cov 2 .Depression was assessed by the Beck Depression Inventory (BDI).

**Results:**

In total, we recruited 30 women in the case group and 30 women in the control group.The average age of the case group was 35.8 ±6.8 years versus an average age of 35.3 ± 6.33 years in the control group. The majority of coronavirus infections were symptomatic (83.3% (n=25)).

The average depression score for the case group was 10.8 ±9.6 corresponding to moderate depression, while the average depression score for the control group was 6.1 ± 6.1 corresponding to mild depression. The presence of depression was more significant in the case group compared to the controls (p=0.003).

**Conclusions:**

COVID-19 is significantly associated with depression, even in mild or pauci-symptomatic clinical forms.

**Disclosure:**

No significant relationships.

